# Comparative mitogenomic analysis and phylogeny of Veneridae with doubly uniparental inheritance

**DOI:** 10.1098/rsob.240186

**Published:** 2024-11-27

**Authors:** Tao Xu, Chuandong He, Xiao Han, Lingfeng Kong, Qi Li

**Affiliations:** ^1^ Key Laboratory of Mariculture, Ministry of Education, Ocean University of China, Qingdao, People’s Republic of China; ^2^ Laboratory for Marine Fisheries Science and Food Production Processes, Qingdao Marine Science and Technology Center, Qingdao, Shandong 266237, People’s Republic of China

**Keywords:** Veneridae, doubly uniparental inheritance, mitochondrial genome, unassigned region, phylogenetic pattern

## Abstract

Doubly uniparental inheritance (DUI) is an atypical animal mtDNA inheritance system, reported so far only in bivalve species, in which two mitochondrial lineages exist: one transmitted through the egg (F-type) and the other through the sperm (M-type). Although numerous species exhibit this unusual organelle inheritance, it is primarily documented in marine and freshwater mussels. The distribution, function and molecular evolutionary implications of DUI in the family Veneridae, however, remain unclear. Here, we investigated 17 species of Veneridae, compared mitochondrial genomes of DUI species and reconstructed their phylogenetic framework. Different sex-linked mitochondrial genomes have been identified in the male gonads and adductor muscles of 7 venerids, indicating the presence of DUI in these species. Analysis of the unassigned regions (URs) of the mitochondrial genome in DUI species revealed that 13 out of 44 URs contained repetitive sequences, with nine being long unassigned regions (LURs). All LURs were capable of forming secondary structures, and most of them exhibited patterns of significant sequence similarity to elements known to have specific functions in the control regions of sea urchins and mammals. The F/M phylogeny showed that DUI venerids exhibit both taxon-specific patterns and gender-specific patterns, with *Gafrarium dispar* experiencing masculinization events.

## 1. Introduction

The only natural and evolutionarily stable heteroplasmic system in Metazoan is doubly uniparental inheritance (DUI) of mitochondria, which was first discovered in the *Mytilus* genus of mussels in 1990 [1–3]. DUI is characterized by the presence of two distinct sex-associated mitochondrial lineages: the female type (F mtDNA) and the male type (M mtDNA). F mtDNA is transmitted through the eggs to all offspring, while M mtDNA, present in sperm, enters all eggs upon fertilization but is exclusively retained and transmitted through male offspring [4,5]. Therefore, these two sex-associated mtDNAs are inherited separately and undergo independent evolution [6]. Genetic analyses suggest that both the F and M lineages undergo rapid molecular evolution compared to typical metazoan mtDNA, with M mtDNA often evolving faster than F mtDNA [7–9]. Conspecific F and M mtDNA exhibit a notable nucleotide sequence divergence, varying from 22% to 39% in mytilids and from 38% to 58% in unionids, resulting in an amino acid sequence divergence of mitochondrial OXPHOS proteins exceeding 50% [10–12]. While the molecular mechanisms underlying DUI remain elusive, certain structural and functional characteristics of M and F mtDNAs, such as sex-specific orf and *cox2* modification (i.e. extensions, insertions and duplications), have been suggested to potentially influence mitochondrial genetics and germline establishment/differentiation [13–15]. Additional sex-specific mitochondrial open reading frames (ORFs), lacking recognizable homologies to other known genes [16], are believed to have originated from the endogenization of viral genes or the duplication and subsequent modification of existing mitochondrial genes and sequences [17–20]. The functions of orf were hypothesized from *in silico* analyses, which support their direct involvement in the DUI mechanism [13,14,17,20,21]. Specifically, F-ORF proteins are suggested to be involved in the regulation of mtDNA replication and/or transcription, and M-ORFs are suggested to prevent the recognition of male-transmitted mitochondria by the degradation machinery [13,17]. Unlike sex-specific orf, the modification of the *cox2* gene appears to be a non-canonical feature of DUI species, and their specific function has not yet been demonstrated. Considering the lack of masculinization coincides with the presence of a unique M genome-specific 3′ extension of *cox2*, known as M*cox2e*, in unionida [22–24], M*cox2e* was inferred as a protective mechanism against sex-switching or beneficial for male reproductive function [4]. However, this pattern is not shared by all DUI species for which sex-specific mtDNAs have been completely sequenced.

To date, DUI has been described in more than 100 bivalve species, including marine mussels within the order Mytilida, various freshwater mussels in the order Unionida, and clams of the orders Venerida and Nuculanoida [25]. Extensive research on DUI remains largely unexplored in most bivalve species, with the exception of mytilids and unionids. Until now DUI has searched in snail species also but this phenomenon remains an exclusive feature of bivalves [26,27]. Conducting extensive mitochondrial surveys across bivalve and other molluscan species is crucial for assessing the prevalence of DUI in molluscs and evaluating its origins. Furthermore, further exploration of the sequence and structural characteristics of DUI species’ mitochondrial genomes can help elucidate the mechanisms underlying the emergence of DUI.

Veneridae (Rafinesque, 1815) is one of the most diverse families of bivalve molluscs, with more than 800 extant species [28]. Until now, within Veneridae (Venerida), only seven species have been identified as DUI species (*Meretrix lamarckii*, *Ruditapes philippinarum*, *Venerupis aspera*, *Macridiscus aequilatera*, *Macridiscus multifariu*s*, Macridiscus donacinus* and *Polititapes rhomboides*), while four other species show an absence of DUI (*Ruditapes decussatus*, *Venus verrucosa*, *Mercenaria mercenaria* and *Eurhomalea rufa*). Here, we sequenced and investigated the presence of DUI in 17 species of the family Veneridae and present the complete or nearly complete M and F mitochondrial genomes of these DUI species. To better characterize the F and M type of mitogenome, we present a comparative analysis of mitogenomes of 11 DUI venerids to highlight both unique features and characteristics shared among different species. Phylogenetic relationships were reconstructed to alleviate the phylogenetic pattern of DUI species in Veneridae.

## 2. Material and methods

### 2.1. Specimen collection

For this work, specimens of the following species were examined: *Meretrix lusoria* (Röding, 1798) (Meretricinae), *Tapes literatus* (Linnaeus, 1758) (Tapetinae), *Tapes conspersus* (Gmelin, 1791) (Tapetinae), *Marcia japonica* (Gmelin, 1791) (Tapetinae), *Paratapes undulatus* (Born, 1778) (Tapetinae), *Cryptonema producta* (Kuroda and Habe, 1951) (Tapetinae), *Anomalodiscus squamosus* (Linnaeus, 1758) (Venerinae), *Cyclina sinensis* (Gmelin, 1791) (Cyclininae), *Meretrix lyrata* (G. B. Sowerby II, 1851) (Meretricinae), *Placamen isabellina* (R. A. Philippi, 1849) (Venerinae), *Antigona chemnitzii* (Hanley, 1845) (Venerinae), *Timoclea marica* (Linnaeus, 1758) (Venerinae), *Globivenus toreuma* (A. Gould, 1850) (Venerinae), *Periglypta puerpera* (Linnaeus, 1771) (Venerinae), *Circe scripta* (Linnaeus, 1758) (Gouldiinae), *Gafrarium dispar* (Röding, 1798) (Gouldiinae), *Gafrarium divaricatum* (Gmelin, 1791) (Gouldiinae), *Dosinia japonica* (Reeve, 1850) (Dosiniinae) and *Saxidomus purpurata* (G. B. Sowerby II, 1852) (Callocardiinae). They were sampled from the Beibu Gulf or purchased at the seafood market around Hainan in May 2023, with the exception of *D. japonica* and *S. purpurata*, which were purchased from the Weihai farmers’ market (Shandong Province, China) in July 2023. The specific collection information of each specimen is listed in electronic supplementary material, table S1. All individuals were dissected while alive, and gonadal content was collected and inspected microscopically, to check sexual maturity and to determine the sex of each specimen. Upon examination, it was determined that exclusively female specimens of *T. marica* and *G. toreuma* were collected.

### 2.2. DNA extraction, and mitochondrial genome assemblies and annotation

Total genomic DNA was extracted from the adductor muscle and gonad tissue, respectively, using TIANamp Marine Animals DNA Kit (Tiangen Biotech Beijing Co.) following the manufacturer’s protocols. Genomic DNA was submitted to Beijing Novogene Technology Co. for library construction and high-throughput sequencing. Sequencing libraries with average insert sizes of approximately 300 bp were prepared. Sequencing of 150 bp paired-end reads was done on the Illumina NovaSeq 6000 platform.

Raw sequence data were trimmed by Trimmomatic 0.36 [29]. Mitogenomes were reconstructed using NOVOPlasty 4.1 [30] and MitoZ v. 2.3 [31]. Protein-coding genes (PCGs) were predicted using both MITOS web server [32] and the Open Reading Frame Finder (https://www.ncbi.nlm.nih.gov/orffinder/) using invertebrate genetic code for mitochondria and alternative start codons, and then followed by manual genome annotation in Artemis [33]. Gene boundaries were examined and manually adjusted by comparison with the previously published venerid mitochondrial genome. Putative tRNA genes were determined using ARWEN [34] and tRNAscan-SE 1.21 [35]. rRNA sequences were identified in BLAST (https://blast.ncbi.nlm.nih.gov/Blast.cgi) and determined their boundaries (i.e. with reference to start and stop codons of adjacent genes).

### 2.3. Characterization of mtDNAs and sequence analyses

The *p*-distances at the amino acid level, nucleotide level and nucleotide base composition were calculated using MEGA 11 software [36]. The skewness was calculated using the following formulae: AT skew = (A − T) / (A + T) and GC skew = (G − C) / (G + C) [37]. The ratios of nonsynonymous and synonymous substitution rates (dN/dS) were calculated by CODEML program in the PAML package [38]. F and M *cox2* sequences were aligned using the T-Coffee Server (https://tcoffee.crg.eu/apps/tcoffee/do:psicoffee) and then graphically edited using DnaSP6 [39]. Transmembrane helices (TMH) were characterized using TMHMM server v2.0 (http://www.cbs.dtu.dk/services/TMHMM/) and Phobius (https://phobius.sbc.su.se/).

Unassigned regions (URs) were assessed for characteristics of control regions as follows. Putative secondary structures were predicted using the mfold webserver [40]. URs were run through Tandem Repeats Finder (https://tandem.bu.edu/trf/trf.html) with basic default parameters to check for tandem repeats [41]. A-T nucleotide content was checked with Geneious [42]. In addition, considering that largest unassigned regions (LUR) were regarded as the prime candidate for the main control region, we conducted a comparison between LUR and well-characterized control regions from sea urchins *Strongylocentrotus purpuratus* (GenBank accession no. NC001453, region 1085–1205) and humans *Homo sapiens* (GenBank accession no. NC001807) [43–48]. The compared elements (including the termination-associated sequence element, considered as the termination site for heavy strand synthesis [49]; the CSB1 conserved sequence block element, one of the three CSBs assumed to contribute to replication and/or transcription [50]; and three binding sites for transcription factors, namely mTF1, mt3 and mt4 [51,52]) are consistent with those employed by [53], which have been confirmed to be involved in the initiation or termination of genome replication or transcription [54].

### 2.4. Phylogenetic analysis

The classification, source data and GenBank accession numbers for all species analysed were listed in electronic supplementary material, table S1. Phylogenetic analyses were conducted using both amino acid (AA) and nucleotide (NT) datasets, comprising 85 sequences representing 44 taxa. Of these, 55 mitogenomes were sourced from GenBank, while 30 mitogenomes were sequenced in the present study. 13 protein-coding genes and two rRNA genes were extracted from the venerid mitogenomes. Amino acid sequences of 13 protein-coding genes (PCGs) were extracted and concatenated in Matrix1. The saturation of nucleotide substitutions for rRNAs and each codon site of PCGs were detected and excluded by DAMBE [55], and then these unsaturation parts were concatenated in Matrix2. Afterward, MAFFT [56] was used to align sequences, followed by Gblocks [57] with default parameters to remove ambiguously aligned positions. Trimmed sequences were concatenated into two different super matrices using FASconCAT [58]. Amino acid sequences of 13 PCGs were extracted and concatenated in Matrix1. The unsaturation nucleotides of 13 PCGs and two rRNA were concatenated in Matrix2.

Phylogenetic trees were reconstructed using both maximum likelihood (ML) and Bayesian inference (BI) analyses. ML analyses were performed using IQ-TREE [59] with the setting of ‘-m MFP’ on each partition. An additional empirical profile mixture model, C60, was also carried out on the AA matrix (Matrix1). All ML analyses were performed with 1000 replicates of ultrafast bootstrapping (-bb 1000). BI was carried out using PhyloBayes MPI v. 1.8c [60] with site-heterogeneous mixture model CAT-GTR models and discarding constant sites (‘-dc’ option). Two independent MCMC chains were running until convergence and convergence was checked with the bpcomp program. Consensus trees were obtained after discarding the first 10% cycles as burn-in. Phylogenetic trees were visualized in FigTree v. 1.4.4 [61].

## 3. Results

### 3.1. Mitochondrial genome organizations

In this study, we investigated the presence of DUI in 17 species of the family Veneridae. The presence of two distinct, sex-linked mitochondrial genomes establishes *Tapes literatus*, *T. conspersus*, *Paratapes undulatus*, *Placamen isabellina*, *Antigona chemnitzii*, *Circe scripta*, *Gafrarium dispar*, *G. divaricatum*, *Dosinia japonica*, *Periglypta puerpera* and *Saxidomus purpurata* as DUI species. Conversely, the absence of mitochondrial heteroplasmy in adductor muscles and gonads confirms the absence of DUI in *Meretrix lusoria*, *Marcia japonica*, *Cryptonema producta*, *Anomalodiscus squamosus*, *Cyclina sinensis* and *Meretrix lyrata*.

Within Veneridae, the mitochondrial genomes that were determined to be incomplete are indicated with an asterisk in electronic supplementary material, table S1. The complete F mtDNAs of 11 newly sequenced DUI species varied in length, ranging from 16 847 bp (*Periglypta puerpera*) to 21 651 bp (*G. divaricatum*), while the complete M mtDNAs ranged from 16 993 bp (*D. japonica*) to 25 197 bp (*G. divaricatum*) (electronic supplementary material, table S1). The difference in mitochondrial length between F and M mtDNA was mostly attributed to differences in the non-coding regions, which ranged from 93 bp (*P. isabelline*) to 2894 bp (*G. divaricatum*) (electronic supplementary material, tables S1,2). For *T. literatus* and *Circe scripta*, an additional contributing factor to the differences is the modification of the *cox2* gene (see below). The newly determined mitogenomes of DUI species, except for the F and M mtDNAs of *T. conspersus T. literatus*, *Paratapes undulatus*, the F mtDNAs of *Circe scripta* and *Antigona chemnitzii*, and the M mtDNAs of *G. divaricatum*, encompass 13 PCGs, two rRNA and 22 tRNA genes. At least one tRNA gene (*trnK*, *trnE*, *trnS1* or *trnM*) duplication was found in above nine newly sequenced mitochondrial genomes of DUI species (electronic supplementary material, table S2). It is worth noting that the M mtDNAs of *T. literatus* has a *rrnS* gene duplication. Six different start codons were observed (electronic supplementary material, table S2), but most protein-coding genes (70.10%) start with ATD initiation codon (where D means A, T, C or G), except for F*cox1* and M*cox3* gene in *Periglypta puerpera* that ends with an incomplete stop codon (T). The AT contents ranged from 64.6% (*Periglypta puerpera*) to 72.7% (*Circe scripta*) in F mtDNAs, while in M mtDNAs ranged from 65.0% (*Paratapes undulatus*) to 73.0% (*Circe scripta*) (electronic supplementary material, table S3). Within the DUI species of venerids, a uniformity in AT contents was observed in F and M mtDNAs, with the most substantial deviation observed in *Periglypta puerpera*, which amounted to 3.2%. All functional genes (PCGs, rRNAs and tRNAs) are encoded on the forward strand, and their nucleotide compositions are strongly skewed away from A in favour of T (except rRNA) and away from C in favour of G. AT skews in rRNAs were irregular, with positive and negative results.

**Figure 1.**
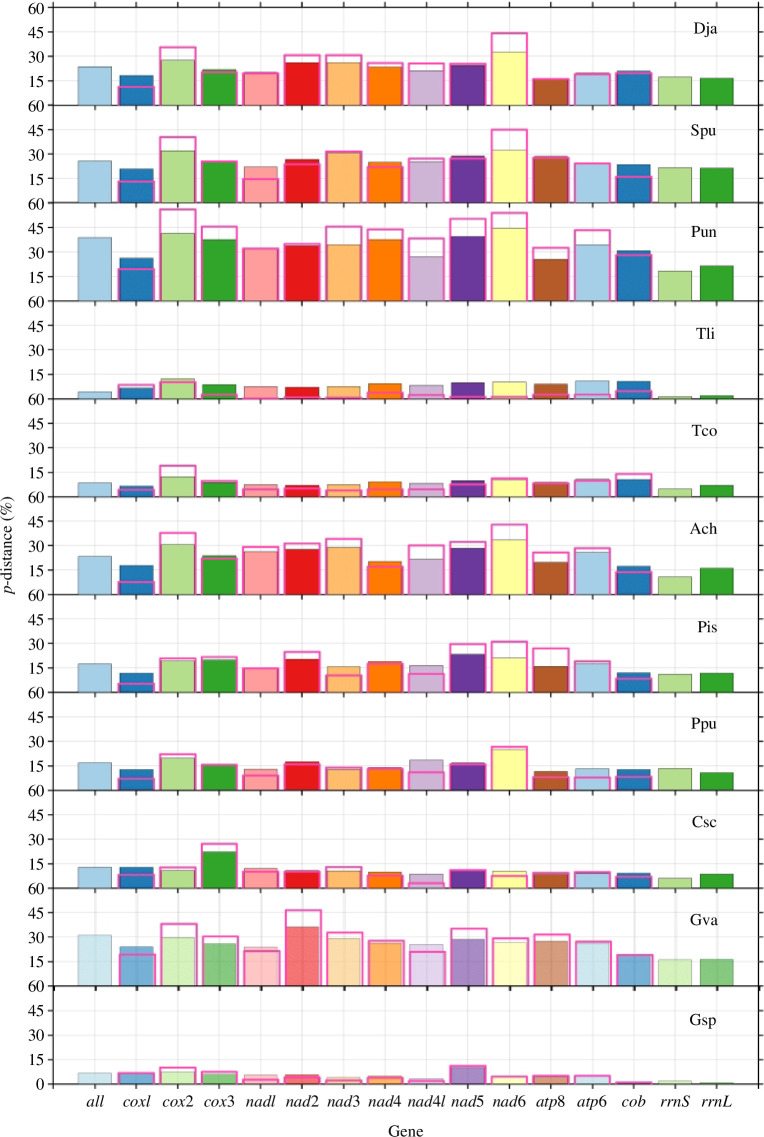
Intraspecific divergence (uncorrected *p*-distances) of the whole sequence, the protein-coding genes (PCGs) and rRNA genes between M and F mtDNAs in 11 newly determined DUI species. Bars filled with a colored background and bordered in black indicate results for nucleotides (NT), while bars not filled with a colour and bordered in rose indicate results for amino acids (AA). The details are recorded in electronic supplementary material, table S4. Dja, *Dosinia japonica*; Spu, *Saxidomus purpurata*; Pun, *Paratapes undulatus*; Tli, *Tapes literatus*; Tco, *Tapes conspersus*; Ach, *Antigona chemnitzii*; Pis, *Placamen isabelline*; Ppu, *Periglypta puerpera*; Csc, *Circe scripta*; Gva, *Gafrarium divaricatum*; Gdi, *Gafrarium dispar*.

**Figure 2.**
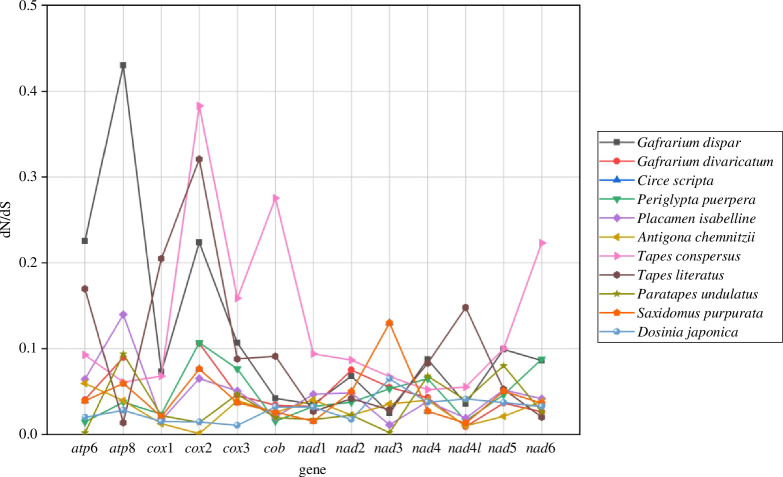
Rates of synonymous and non-synonymous substitutions within mitochondrial PCGs in newly sequenced DUI for Veneridae.

Genetic distances of mitogenome and individual genes between F and M mtDNAs were provided in figure 1 and electronic supplementary material, table S4. The pairwise genetic distances ranged from 4.2% (*T. literatus*) to 38.7% (*Paratapes undulatus*) for the complete mitogenome and from 1.4% (*G. dispar*) to 21.4% (*Saxidomus purpuratus*) for all rRNAs. For all PCGs, the results showed the average *p*-distance range from 4.4% (*T. literatus*) to 34.6% (*Paratapes undulatus*) for nucleotide (NT) and from 3.1% (*T. literatus*) to 42.0% (*Paratapes undulatus*) for amino acid (AA) in Veneridae DUI species. Genetic distances of individual genes showed that the level of conservation is higher for *rrnS*, *rrnL* and *cox1*. The non-synonymous (dN) and synonymous (dS) substitution rates were calculated to reflect the evolutionary dynamics of protein-coding sequences across F and M mtDNAs of DUI species. The dN/dS ratios of 13 PCGs of all DUI species were less than 1, ranging from 0.001 to 0.43, indicating that these genes were under negative or purifying selection (figure 2).

### 3.2. *Cox2* modification in F and M venerid genomes

**Figure 3.**
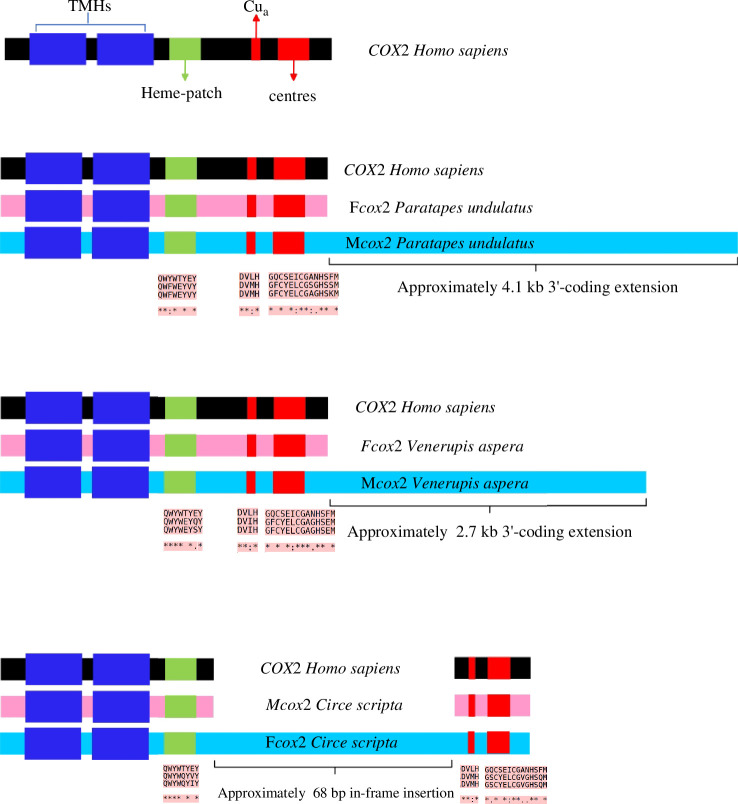
F*cox2* and M*cox2* structural features in *Paratapes undulatus*, *Venerupis aspera* and *Circe scripta*. For further details on *V. aspera*, see [62]. For the sequence alignments of the ‘heme-patch’ regions and Cua centres, identical amino acids are indicated by an asterisk (modified from [63]).

Annotations indicate that, except for *Paratapes undulatus*, *Circe scripta* and *V. aspera*, there are no modifications of the *cox2* gene in other DUI species. The M*cox2* of *Paratapes undulatus* features an approximately 4100 bp 3′-coding extension, while an insertion of 86 codons within the *cox2* gene has been identified in the F mtDNA of *Circe scripta*. However, additional TMHs, either in insertions or extensions, are not detectable in *Paratapes undulatus* M*cox2*, nor in *Circe scripta* F*cox2* (figure 3; electronic supplementary material, figure S1).

### 3.3. Unassigned regions in F and M venerid mitochondrial genomes

Unassigned regions of 1855 bp (*Periglypta puerpera*) to 5206 bp (*G. divaricatum*) (F genomes), and 1737 bp (*S. purpurata*)to 8055 bp (*G. divaricatum*) (M genomes), are found in DUI venerid genomes, encompassing 9.7% (*S. purpurata*) to 32.0% (*G. divaricatum*) of the total length of the mitochondrial genomes (electronic supplementary material, table S5). Putative secondary structures were located within all principal URs (>150 nucleotides in length) in both F and M mtDNAs (table 1). However, repeats were found exclusively in five F-type and four M-type complete mtDNAs, totaling 13 URs, of which 9 were LUR. Only in *Circe scripta* and *Paratapes undulatus*, both F and M URs have consecutive repeats. The comparison between LUR and well-characterized control regions from the sea urchins *Strongylocentrotus purpuratus* shows similarities between the sea urchin control region and the LUR (electronic supplementary material, figure S2). The *S. purpuratus* control region contains a string of 20 Gs, and a string of similar length (ranging from 18 to 23 bp) with approximately 80% in G is present in most LURs. Besides, similar sequence was not found in the F LUR of *G. dispar*, M LUR of *Circe scripta*, and F LUR and M LUR of *T. literatus*. However, a similar sequence of this string was found in the second longest unassigned region of *Circe scripta*, and it exactly matches the sequence in the F LUR of *Circe scripta*. In addition, the identification of motifs showing significant sequence similarity (>50%) with elements known to be involved in the initiation or termination of genome replication or transcription in the mammalian control region was possible in DUI Veneridae LUR (electronic supplementary material, figure S2).

**Table 1.** General characteristics of unassigned regions. The F genomes of *T. literatus* have incomplete sequences in the unassigned region and are therefore not included.

DUI species	UR	length (bp)	A-T content (%)	no. of repeats units	copy no. of repetitive elements	other characteristics
*Gafrarium dispar* F	*nad1-nad2*	542	77.5	0	/	stem loop
	*nad5-trnF*	1001	69.5	0	/	stem loop/hairpin structure
*Gafrarium dispar* M	*nad1-nad2*	558	78.0	2	2+2.4	stem loop/hairpin structure
	*nad5-trnF*	3191	72.1	1	2	stem loop/hairpin structure
*Gafrarium divaricatum* F	*nad5-trnK*	3157	66.8	0	/	stem loop
*Gafrarium divaricatum* M	*nad4-cox2*	1606	64.5	0	/	stem loop/hairpin structure
	*cox2-trnP*	1613	67.9	0	/	stem loop
	*nad5+trnF-trnK*	2806	67.5	0	/	stem loop/hairpin structure
*Circe scripta* F	*nad5-trnF*	2031	75.0	3	1.9+21+1.9	stem loop/hairpin structure, (A)n, (T)n
	*trnQ-rrnS*	183	77.5	1	14.5	stem loop/hairpin structure
	*cox3-cox1*	246	81.7	0	/	stem loop/hairpin structure
*Circe scripta* M	*nad5-trnF*	1578	70.9	0	/	stem loop/hairpin structure
	*trnQ-rrnS*	167	77.2	1	17.5	stem loop/hairpin structure
	*trnS-trnS*	2104	76.1	1	2.1	stem loop/hairpin structure, (T)n
*Placamen isabelline* F	*trnY-trnF*	1324	71.5	2	8+3.8	stem loop/hairpin structure, (A)n,
	*nad5-trnD*	446	68.6	0	/	stem loop/hairpin structure
	*trnD-trnY*	163	79.3	0	/	stem loop
*Placamen isabelline* M	*trnY-nad6*	1303	68.0	0	/	stem loop/hairpin structure
	*trnA-cox1*	536	69.4	0	/	stem loop/hairpin structure
*Antigona chemnitzii* F	*nad5-nad4L*	1438	68.8	0	/	stem loop/hairpin structure
	*atp8-nad4*	391	67.8	0	/	stem loop
*Antigona chemnitzii* M	*nad5-nad4L*	3110	72.5	0	/	stem loop/hairpin structure
	*atp8-nad4*	368	69.6	0	/	stem loop
*Periglypta puerpera* F	*nad3-nad5*	257	67.7	0	/	stem loop
	*nad5-trnM*	426	71.10	0	/	stem loop
	*nad4L-cox2*	479	58.2	0	/	stem loop
*Periglypta puerpera* M	*nad3-nad5*	373	76.10	0	/	stem loop
	*nad5-trnM*	1657	61.20	2	2.1+1.9	stem loop/hairpin structures, (G)n
	*trnM-nad4L*	331	73.1	0	/	stem loop
*Tapes literatus* F	/	/	/	/	/	/
*Tapes literatus* M	*nad5-rrnS*	754	60.7	0	/	stem loop
	*rrnS-atp8*	517	72.9	0	/	stem loop
*Paratapes undulatus* F	*nad5-atp8*	1532	64.9	1	2	stem loop/hairpin structure
	*cox3-rrnS*	241	69.3	0	/	stem loop
*Paratapes undulatus* M	*nad5-atp8*	1669	66.2	1	3	stem loop
	*cox3-rrnS*	780	71.2	2	2+2	stem loop/hairpin structure
	*cox2-trnP*	786	71.2	0	/	stem loop/hairpin structure
	*rrnL-atp6*	278	71.6	0	/	stem loop
*Tapes conspersus* F	*nad5-atp8*	1874	64.6	0	/	stem loop/hairpin structure
*Tapes conspersus* M	*nad5-atp8*	1705	64.8	0	/	stem loop/hairpin structure
*Saxidomus purpuratus* F	*nad4L-trnI*	1182	70.3	0	/	stem loop
	*trnR-trnF*	1253	65.0	2	2.9+5.2	stem loop
*Saxidomus purpuratus* M	*trnR-trnF*	1015	66.1	0	/	stem loop
*Dosinia japonica* F	*trnI-trnY*	2000	72.9	1	1.9	stem loop/hairpin structure
*Dosinia japonica* M	*trnI-trnY*	1444	76.0	0	/	stem loop/hairpin structure

### 3.4. Phylogenetic analysis

The detection of substitution saturation showed that all third codon positions of PCGs, second codon positions of *cox2*, *nad4l*, *nad6* and *atp8*, and first codon positions of *cox2*, *nad2*, *nad3*, *nad4*, *nad4l*, *nad5*, *nad6*, *atp6* and *atp8* were saturated. Phylograms derived from different methods and matrices had almost identical topologies except certain internal nodes (electronic supplementary material, figure S3).

**Figure 4.**
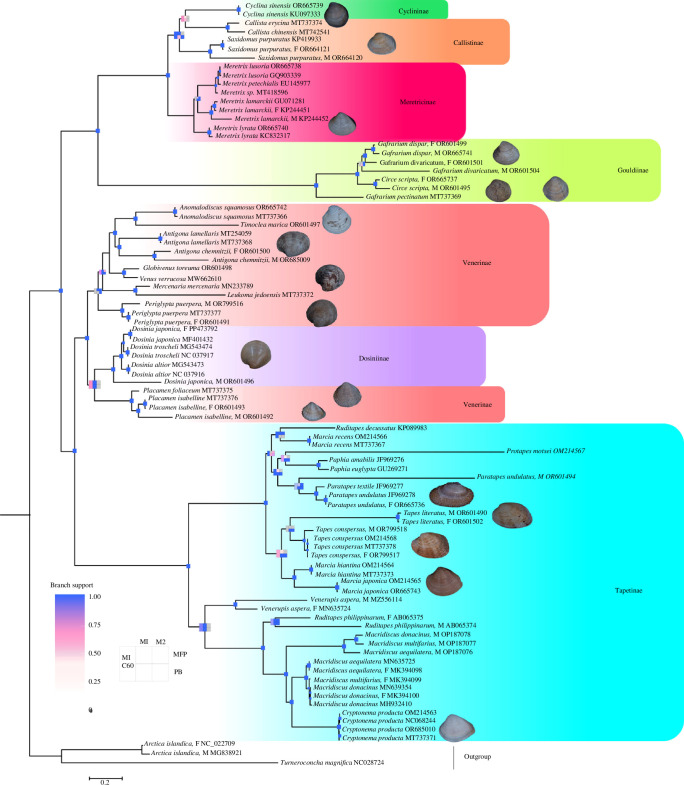
Mitogenome phylogeny of Veneridae, reconstructed with multiple methods. The support values of nodes are mapped onto a matrix, with colors transitioning along a gradient from 0 (white) to 100% (blue) on a continuous scale. Blue indicates full support at 100%, while white indicates topologies not supported by the representative tree. Nodes marked with blue dots signify complete support across all methods. M1–M2 indicates Matrix1–Matrix2; C60 and MFP represent the corresponding models implemented in IQ-Tree; PB, PhyloBayes.

In *D*. *japonica*, *Paratapes undulatus* and three *Macridiscus* species, the M and F clades are reciprocally monophyletic, meaning that the F sequences of different species cluster together as do the M sequences, exhibiting a ‘gender-joining’ phylogenetic pattern (figure 4). *M. lamarckii*, *Antigona chemnitzii*, *P. isabelline*, *T. literatus*, *T. conspersus* exhibit a ‘taxon-joining’ pattern, respectively, in which the F-type and M-type mitogenomes sequences cluster together in a monophyletic group. It appears that *Circe scripta*, *Periglypta puerpera*, *V. aspera*, *S. purpurata* and *R. philippinarum* demonstrate a ‘taxon-joining’ pattern. However, further validation is required by incorporating additional species from the same genus or closely related taxa into the existing phylogenetic tree. Notably, the F genome of *G. divaricatum* forms a clade with the M and F mtDNAs of *G. dispar*, while M genome of *G. divaricatum* is recovered as their sister taxon.

### 3.5. Gene order analyses

The gene arrangement between the F and M mtDNAs of DUI species is highly conservative (electronic supplementary material, figure S4). The M mtDNA of *G. divaricatum* and the F mtDNA of *Circe scripta*, *Antigona chemnitzii*, and *P. isabelline* exhibit replication or the absence of certain tRNAs. Rearrangements in DUI venerid species were restricted to tRNA genes, the most mobile genes within the animal mt genome [64,65]. Specifically, gene transpositions occurred in *trnM* of *Antigona chemnitzii*, *trnD* of *D. japonica* and *trnQ* of *Paratapes undulatus*. Additionally, the M mtDNA of *T. literatus* and the F mtDNA of *R. philippinarum* exhibit replication of *rrnS* and *cox2*, respectively. Extensive rearrangements were only observed in *Macridaiscus*. Specifically, the M mtDNA of *Macridaiscus* only preserves the clusters *trnH-trnE-trnS2-atp6-nad3-nad5-trnY-trnM-trnD-nad6*, *trnL1-nad1-nad2-nad4L-trnI-cox2-trnP-rrnL-atp8-nad4*, *trnA-trnS1-cox3-rrnS*, *trnF-trnW-trnR*, *trnL2-trnG-trnQ*, *trnK-trnV* and *trnT-trnC* when compared with the gene order of the F mtDNA.

## 4. Discussion

### 4.1. The quest for DUI in venerid species

In this study, the count of DUI species within Veneridae was expanded from seven to 18. Additionally, it was confirmed that six venerid species lack DUI, increasing the total number of venerid species without DUI to ten. Notably, GenBank data, with accession numbers AB040833 (M1), AB040834 (M2) and AB040835 (F), suggest the potential occurrence of DUI in the species *Cyclina sinensis*. In the phylogenetic tree reconstructed based on the concatenated *cox1-rrnS* dataset, AB040833 was sister to AB040834+AB040835 [66]. In this study, heteroplasmic mitogenome was not observed in male *Cyclina sinensis* with mature gonads. Moreover, the *cox1* sequence of the male gonad mitochondrial genome shares an identity of >99% with AB040833, as well as with other *cox1* sequences of *Cyclina sinensis* in NCBI excluding AB040834 and AB040835. This suggests the absence of DUI in *Cyclina sinensis*. As a result, the reliability of these *cox1* data requires further investigation.

### 4.2. Comparison of the DUI genome features of the two sex-linked genomes

The nucleotide genetic distance between the F and M mtDNA of DUI species in mytilids ranges from 22% to 39% and 38% to 58% in unionids [10–12,67]. We compared previously published complete mitochondrial genomes in all DUI venerids, revealing an intraspecific nucleotide divergence between M-type and F-type mitogenomes ranging from around 16.6% (*M. lamarckii*) to 48.2% (*M. donacinus*) (see electronic supplementary material, table S6). The nucleotide divergence of newly determined DUI venerids generally falls within this range, with exceptions noted for *G. dispar* (6.6%), *Circe scripta* (12.6%), *T. conspersus* (8.3%) and *T. literatus* (4.2%).

Notably, the two mitochondrial lineages highlighted in *G. dispar*, *T. conspersus* and *T. literatus* exhibit relatively low levels of sequence divergence, ranging from 6.4% to 7.1% for *cox1* and from 0.8% to 7.0% for *rrnL. Arctica islandica* (Venerida, Arcticidae) emerges as the least genetically divergent DUI species known, with a distance of 7.6% between the F and M type mitochondrial genomes, and genetic distances between the *cox1* and *rrnS* full sequences ranging from 5.4% to 8.0% [68]. Of the studied DUI venerids, *cox2* modifications were exclusively found in *P. undulatus* and *Circe scripta*. In *Paratapes undulatus*, this extension, if translated, means that the M*cox2* gene would be 1856 bp-long and would therefor encode the second longest *cox2* protein in the animal kingdom. The longest *cox2* protein, with a >4.5 kb in-frame insertion (i.e. 1893 amino acids), was found in M*cox2* of DUI species *Scrobicularia plana* [63]. The alignment of amino acids from *Homo sapiens COX2*, *S. plana* F*cox2* and *S. plana* M*cox2* reveals that the insertion occurs between the crucial ‘heme-patch’ region [63]. This region contains crucial residue Trp, which are essential for electron entry from Cytochrome C, and also houses the initial Cua-binding centre [63] . A similar insertion occurred in F*cox2* of *Circe scripta*. Moreover, analysis of the mitochondrial genome sequences in NCBI unveiled a specific 3′ extension (approx 2.7 kb) in the M*cox2* gene of *V. aspera*, a species previously known for DUI. In Veneridae, other DUI species have also been reported to exhibit modification of the *cox2* gene. *M. lamarckii* genome presents an in-frame insertion of 300 bp in M*cox2* [69]. Additionally, *R. philippinarum* contains a duplicated of the F*cox2* gene with a 3′ extension (GenBank accession no. AB065375; unpublished GenBank annotation mentioned in [70]). However, whether the *cox2* modifications observed in DUI Veneridae are functional and what the specific functions are need to be further explored. With the discovery of more DUI species, it may become possible to explain the relationship between these structural variations in the *cox2* gene and DUI, as well as the absence of *cox2* gene modifications in *Mytilus* and many DUI species of the Veneridae.

### 4.3. Unassigned regions in F and M venerid mitochondrial genomes

In newly sequenced DUI of venerid species, the proportion of unassigned sequences in F and M venerid genomes ranges from 9.7 to 32%. Similar results were observed in *R. philippinarum*, with 15.8% of sequences unassigned in F mtDNA and 21.3% unassigned in M mtDNA [21] . In unionids and mytilids, the non-coding region constitutes approximately 10% of the total mitochondrial genome length in both F and M genomes [21,71–73]. This may be due to the fact that Veneridae has undergone more significant mitochondrial genome rearrangements, with tandem duplication-random loss leading to an increase in unassigned regions [64,74–76].

Although secondary structures were located within all major URs of all available complete F and M-type mtDNAs, identifying a main control region similar to other metazoan mitochondrial genomes remains challenging. In the complete F and M mtDNA of the 11 DUI venerid species, only five F-type and four M-type mitogenome contain repeats. The loss of repetitive sequences in URs also occurs in hermaphroditic freshwater mussels, while in the URs of dioecious freshwater mussels and marine mussels, repetitive sequences are consistently present [21,53,77,78]. The loss of repetitive sequences in the UR in hermaphroditic freshwater mussels has been hypothesized to be associated with shifts in reproductive strategy and corresponding changes in the processes of germ line, gonadal tissue and gamete development [78]. Currently, it is not clear why there is a widespread loss of repetitive sequences in Veneridae URs. To further search the control region, we compared the LUR with well-characterized control regions of sea urchins *S. purpuratus* and humans *H. sapiens*. While this method has not yielded successful results in defining potential regulatory elements in unionid genomes [21], it is an important piece of evidence for determining the control region of *Mytilus* [21,53]. A string of 20 Gs, similar to the sea urchin control region, was found in third domain of *Mytilus* LUR [53]. A similar string with 80% in G was found in almost LUR sequence of venerids. Remarkably, the string is absent in the M LUR (trnS-rrnS) of *Circe scripta* but is found in nad5-trnF, located at the same genomic position as the F LUR. Moreover, the string of Gs in the M *nad5-trnF* is identical to the sequence in the F LUR of *Circe scripta*. Additionally, the M *trnS-rrnS* region features a large open reading frame (ORF), constituting the majority (95%) of the unassigned sequence. Despite the presence of repetitive sequences and putative secondary structures in the M *trnS-rrnS* region, it seems that *nad5-trnF* functions as the control region in M *Circe scripta*. Apart from the string, a conserved motif, TATATATAA, similar to the sea urchin control region and potentially representing a bidirectional promoter or a binding site for transcription termination factors, was identified in the LUR of *Mytilus* [53]. However, the conserved element could not be found in Veneridae. As with *Mytilus*, we found motifs in Veneridae with remarkable similarity to mammalian control regions that are involved in genome replication or the initiation or termination of transcription.

### 4.4. Phylogenetic analysis

In the F/M phylogeny of unionids, there is a strict gender-joining pattern, where the F genomes from all species, genera and even subfamilies form one cluster, while all the M genomes form another [23,25,79–81]. While the possibility of an F-to-M reversal cannot be definitively ruled out without an extensive population-based survey of the paternally inherited genome, the current findings strongly suggest that the non-fixation of masculinized genomes appears almost certain [10]. It has been proposed that this may be associated with *cox2* extension, consistently present in the M genomes of unionids [10,22]. In Veneridae, although three DUI species belonging to *Macridiscus*, namely *M. donacinus*, *M. donacinus* and *M. aequilatera,* exhibit gender-joining patterns similar to Unionidae, the *cox2* gene modifications are absent in the mitochondrial genomes of these three species. For species *Circe scripta*, *Periglypta puerpera*, *V. aspera* and *R. philippinarum*, the absence of both mitochondrial genome sequences within the same genus and identification of DUI hinders our inference about the origin of DUI. Therefore, it is necessary to conduct extensive DUI investigation studies.

The phylogeny pattern of *G. divaricatum* resembles that of *M. californianus*, with its M genome branching away from the cluster of other genomes [10]. According to the hypothesis favoured by previous studies [7,80], *M. edulis*, *M. trossulus* and *M. californianus* originated from a single event of DUI, with masculinization occurring in the common ancestor of *M. edulis*/*M. trossulus*. Combining phylogenetic relationships with the genetic distance observed between the F and M mitochondrial genomes in *G. dispar*, we deduce the occurrence of masculinization in *G. dispar*, indicating that the contemporary M genomes of *M. edulis* and *M. trossulus* are descendants of this masculinized genome.

## 5. Conclusions

In this study, a heterogeneous mitochondrial genome evidenced the presence of DUI in *T. literatus*, *T. conspersus*, *Paratapes undulatus*, *Placamen isabellina*, *Antigona chemnitzii*, *Circe scripta*, *G. dispar*, *G. divaricatum*, *D. japonica*, *Periglypta puerpera* and *S. purpurata*. The absence of two distinct, sex-linked mitochondrial genomes establishes *Meretrix lusoria*, *Marcia japonica*, *Cryptonema producta*, *Anomalodiscus squamosus*, *Cyclina sinensis* and *Meretrix lyrata* as non-DUI species. *Cox2* gene modifications in DUI venerids specifically involve insertions, duplications and extensions of the *cox2* gene. While not present in some DUI species such as Unionidae, this feature appears to be more prevalent than in Mytilidae. In the mitochondrial DNA of DUI species within Veneridae, a typical control region appears to be absent—LUR in the majority of DUI species lacks repetitive sequences. However, the potential control region sequences show significant sequence similarity to elements found in sea urchin and mammalian control regions, which are known for their involvement in genome replication, or initiation and termination of transcription. The phylogenetic pattern of DUI Veneridae is a mixed mode, encompassing DUI species clustering based on both the origin species of the genomes (taxon-joining pattern) and the mode of transmission (gender-joining pattern).

## Data Availability

The full-length sequences of all newly sequenced mitochondrial genomes have been deposited in GenBank and are accessible through GenBank accessions: OR601493, OR601490–OR601502, OR601504, OR664120–OR664121, OR665736–OR665737, OR665739–OR665743, OR685009–OR685010, OR799516–OR799518 and PP473792. Supplementary material is available online [82].
